# Incidence of Adverse Perinatal Outcomes among Women Exposed to Maternal Near-Misses in Arsi Zone in Ethiopia: Prospective Cohort Study in 2022

**DOI:** 10.1155/2024/6560652

**Published:** 2024-03-21

**Authors:** Wogene Morka, Getu Megersa, Elias Bekele, Abdi Deksisa

**Affiliations:** Department of Midwifery, College of Health Sciences, Arsi University, Asella, Ethiopia

## Abstract

**Background:**

Exposure to maternal near-misses has a massive effect on adverse perinatal outcomes. Hence, investigating the effect of maternal near-misses on perinatal outcomes can aid in the reduction of perinatal morbidity and mortality. The study is aimed at assessing the incidence of adverse perinatal outcomes among women exposed to maternal near-misses at Arsi Zone public hospitals in Ethiopia in 2022.

**Method:**

The study included a prospective cohort of 335 women at Arsi Zone public hospitals from December 2021 to June 2022. Women who were admitted for management of pregnancy were followed. The exposed group was women with maternal near-misses screened based on disease-validated criteria. The nonexposed group was made up of women who delivered without complications. Trained data collectors used pretested, structured questionnaires to collect data from women. Pertinent data was also extracted from the clients' logbooks. Data was transferred from EpiData version 3.1 to SPSS version 25 for analysis, logistic regression was computed, and 95% confidence intervals were declared at a *p* value of 5% significance level.

**Result:**

The incidence of adverse perinatal outcomes was higher in the exposed women than in the nonexposed women (56% versus 16%). Contrasted with the nonexposed, women exposed to maternal near-misses had a higher incidence of stillbirth (22% vs. 0.5%), low birth weight (13% vs. 3%), and preterm birth (12% vs. 2%). After adjusting for confounders, exposed women had a twofold increased risk of adverse perinatal outcomes compared to nonexposed women. Delivery mode, delay in seeking care, transport mode, and delay in receiving treatment were the risk factors for negative pregnancy outcomes.

**Conclusion:**

In exposed women, a higher incidence of adverse perinatal outcomes was linked to aforementioned risk factors. Evidence-based practice intended to decrease delays in providing maternal care services does indeed improve perinatal outcomes.

## 1. Introduction

Maternal near-miss and its adverse effects on perinatal outcomes are the major public health challenge, despite the continuing efforts to improve the availability and quality of obstetric care services [1]. The World Health Organization (WHO) defines maternal near-miss as “a woman who nearly died but survived a complication during pregnancy, childbirth, or 42 days after termination of pregnancy” [2, 3]. Any conditions affecting the mother's health during pregnancy, labour and delivery, and postnatal period have a direct impact on the infant's well-being [4, 5]. This is due to the close interrelationships of maternal and neonatal health [4, 6, 7]. Failure to improve birth outcomes by 2035 will result in an estimated 116 million deaths, 99 million survivors with disabilities, and 52 million stillbirths [5].

Evidence from the literature revealed a high magnitude of maternal near-misses, such as 34.4% in Arsi Zone [8], 4.97% in Western Ethiopia [9], 92% in Eastern Ethiopia [10], 59.2% in Jimma [11], 23.3% in Amhara [12], and 33.3% in Southern Ethiopia [13], existing in various parts of Ethiopia.

The biggest drivers of adverse perinatal outcomes are complications during pregnancy, labour, and delivery. According to the report from a study conducted in Addis Ababa, Ethiopia, the risk of adverse perinatal outcomes was five times higher in women exposed to maternal near-misses than in women who were not exposed. These complications are strongly associated with the occurrence of negative perinatal outcomes, such as stillbirth, low birth weight, preterm birth, and birth asphyxia [14, 15]. Studies to date showed that adverse perinatal outcome is a common public health problem in developing countries like Ethiopia [16–20]. For instance, a study done in Nigeria reported that the occurrence of maternal near-miss was linked to low birth weight, stillbirth, and birth asphyxia [21].

Delays are significant determinant of maternal and newborn health; however, prior research is overlooked [14, 16, 17]. The extent of negative perinatal outcomes and risk factors in the research environment remains largely unknown, along with scant knowledge on the conditions that contribute to such consequences. Thus, this study is crucial for comprehending the area of interventions to improve birth outcomes. It also fills the knowledge gap and provides trustworthy evidence for consumers at any levels. Therefore, the study is aimed at investigating the incidence of adverse perinatal outcomes among women exposed to maternal near-misses at Arsi Zone public hospitals in Ethiopia.

## 2. Methods and Materials

### 2.1. Study Setting, Design, and Period

A prospective cohort study design was carried out from December 2012 to June 2022 at Arsi Zone public hospitals. Arsi Zone is in Oromia Reginal State; its administrative town is Asella. The zone has seven district hospitals and one teaching and referral hospital; during the data collection period, one hospital was closed for COVID-19 treatment, and the remaining hospitals provide obstetric care, including antenatal care, labour and delivery, and postnatal services. The zone's population is estimated to be 2,637,657, of which 1,314,233 were women, according to a report from the 2007 census.

### 2.2. Source Population

The source population includes all women who were admitted to public hospitals during pregnancy, labour, and delivery and/or within 42 days of delivery or termination of pregnancy.

### 2.3. Study Participants

Sampled women who were managed at participating hospitals during pregnancy, labour, and delivery and postnatal care takers were the study participants. It consisted of a double population group.

*Exposed women:* women with potential life-threatening conditions during pregnancy, labour, and delivery and/or within 42 days of delivery or termination of pregnancy.

*Nonexposed women:* women with normal labour who deliver through spontaneous vaginal delivery.

### 2.4. Group Selection, Enrollment, and Exclusion Principles

Women who met at least one of the WHO criteria [2] and experienced maternal near-miss events were classified as the exposed group. On the other hand, women who gave birth without complications and were unable to meet at least one of the WHO criteria were assigned to nonexposed group. The nonexposed group was chosen based on age interval category and delivered on the same day near-miss events occurred. Women who had abortions or ectopic pregnancies or undelivered were excluded from the study because they did not have viable fetuses to assess perinatal outcomes. Women who gave birth at another health facility were also excluded due to the difficulty of determining perinatal outcome status.

### 2.5. Sample Size and Sampling Technique

The sample size for the cohort study, the double population proportion, was calculated using Epi Info 7 software. The following parameters were used to estimate the sample size, 95% confidence interval, study power 80%, exposed to nonexposed ratio 1 : 2, prevalence of outcome in nonexposed group 1.7%, and prevalence of outcome in exposed group 10% [20], and a 10% loss rate was added. With 223 women in the nonexposed group and 112 women in the exposed group, the minimum required sample size was 335. Based on the three-month average number of client flows, the sample size was distributed proportionately among the four hospitals: Asella Teaching and Referral Hospital (1233), Kersa Hospital (667), Abomsa Hospital (500), and Arsi Robe Hospital (325). Maternal near-miss cases were identified using the WHO disease-validated criteria, such as obstructed labour (uterine rupture, impeding rupture like prolonged labour with previous C/S, and emergency C/S), hemorrhage (severe obstetric hemorrhage leading to shock, emergency hysterectomy, coagulation defects, and/or blood transfusion of at least one unit), pregnancy-induced hypertension disorders (severe preeclampsia, eclampsia), sepsis (septic abortion, infections including hypothermia or hyperthermia or clear source of infection, and clinical signs of septic shock), and severe anemia (including low hemoglobin < 6 g/dL or clinical signs of severe anemia in woman without hemorrhage) [2].

### 2.6. Study Variables

*Dependent variables:* incidence of adverse perinatal outcomes.

*Independent variables:* maternal near-miss, sociodemographic factors, reproductive health-related factors, preexisting medical factors, and accessibility to reproductive health services.

### 2.7. Operational Definitions

*Maternal near-miss*: a pregnant or recently delivered woman who nearly died but survived a complication during pregnancy, childbirth, or 42 days after termination of pregnancy [2].

*Adverse perinatal outcome:* the presence of either one or more of the following, stillbirth, low birth weight, preterm birth, and birth asphyxia, in women with and without maternal near-miss.

### 2.8. Data Collection Tools

Standard questionnaires and data extraction tools adapted from previous studies and slightly modified after a thorough review of the literature were used [2, 9, 14, 22, 23]. The questionnaires were contextualized to collect information on each respondent's demographics, reproductive health and obstetric history, and current and preexisting medical history. Information about women and perinatal status was further abstracted using data extraction tools from medical records and hospital management information systems.

### 2.9. Data Collection Procedure

Women who fulfilled the study's inclusion criteria were enrolled during admission and followed by data collectors during their pregnancies, deliveries, and postpartum periods in order to assess the status of perinatal outcomes. Information about the mother's sociodemographic characteristics, reproductive history, obstetric and medical history using interviewer-administered questionnaires, and her status as a near-miss using WHO-validated disease criteria was collected during enrollment. After delivery, data regarding the mode of delivery and the adverse perinatal outcomes—such as stillbirth, birth weight, preterm birth, and birth asphyxia—were obtained from medical records. Two nurses and six midwives with BSc degrees took part in the data collection procedure. The principal investigator oversaw and coordinated every aspect of the data collection process, with field supervisors deployed accompanying data collectors providing close supervision and verification of completed questionnaires.

### 2.10. Data Quality Control

Language experts translated the questionnaire from English to the local language and vice versa to ensure consistency. The tools were pretested (5% of the sample size) at another hospital to ensure validity and reliability. A one-day training on ethical principles and how to screen, follow, and collect data from women was given to data collectors and supervisors. The data collectors completed and signed every questionnaire, which was later checked and signed by supervisors. The filled questionnaire was safely stored in an appropriate location. Supervisors oversaw the data collection procedures daily.

### 2.11. Data Processing and Analysis

After checking for completeness, data was entered into the EpiData 3.1 version. It was cleaned and then exported to SPSS version 25 for further analysis. The chi-square test was used to determine whether there is a statistically significant difference in selected categorical variables between the exposed and the nonexposed women. Adverse perinatal outcome was dichotomized into yes and no. To examine the relationship between dependent and independent variables, a bivariate analysis was performed, and an odds ratio with 95% confidence interval was computed. To depict a risk factor associated with the dependent variable, all variables with a *p* value of 0.25 in the bivariate analysis were included in the binary logistic regression for further analysis. The statistical significance level was set to a *p* value of 0.05.

### 2.12. Ethical Consideration

The study proposal was first approved by the College of Health Sciences Arsi University Ethical Review Committee, and ethical clearance was obtained with the protocol number A/CHS/RC/60/2022 dated January 2022. Permission letters were obtained from the Arsi Zone Health Bureau. A letter of support was sent to all concerned bodies. After explaining the purpose of the study, duration, potential risks, and benefits, study participants provided verbal informed consent.

## 3. Result

**Figure 1:**
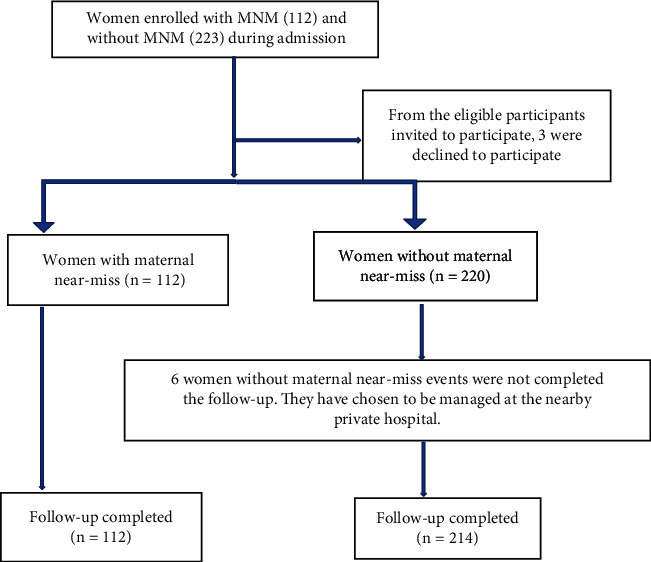
Diagrammatic representation of the follow-up process.

From December 2021 to June 2022, 7455 women were screened; 335 of these were enrolled as eligible, followed, and of those, 100% of the exposed group and 96% of the nonexposed group completed the follow-up processes. Three of the women declined to participate in the study, and six of the women preferred to receive care at the nearby private hospital (Figure 1).

### 3.1. Sociodemographic and Reproductive Health-Related Characteristics

Women exposed to maternal near-misses were more likely to reside in rural areas (*p* < 0.001), attained a primary educational level (*p* = 0.049), have had more than five births (*p* = 0.004), and have no history of ANC contact (*p* = 0.039), than nonexposed women. Absence of ANC contact in the current pregnancy (*p* = 0.039), fewer than four ANC contacts (*p* < 0.001), delay in seeking care (*p* < 0.001), delay in arriving at hospital (*p* < 0.001), delay in receiving treatment (*p* < 0.001), and mode of transport (*p* < 0.001) were the factors significantly linked to the exposed than the nonexposed women (Table 1).

### 3.2. Incidence of Adverse Perinatal Outcomes

Among a total of 326 women delivered during the study period, 29.8% (95% CI: 24.8-35) had a variety of adverse perinatal outcomes. Women exposed to maternal near-miss had a considerably greater incidence of adverse perinatal outcomes than the nonexposed women, 56% versus 16% (*p* < 0.001). Babies born to exposed women were far more likely to be stillborn (*p* < 0.001), underweight (*p* < 0.001), and born prematurely (*p* < 0.001) than babies born to nonexposed women. Even though it was not statistically significant, birth asphyxia was less common in women exposed to maternal near-misses than in the nonexposed women (*p* = 0.605) (Table 2).

### 3.3. Risk Factors

In a logistic regression analysis model, the association between maternal near-misses and adverse perinatal outcomes remained significant after controlling for confounders such as mode of transport, mode of delivery, delay in seeking care, and delay in receiving treatment. Women exposed to maternal near-misses had twice the likelihood of developing poor pregnancy outcomes as women who were not exposed to maternal near-misses (AOR = 2.25, 95% CI: 1.10–4.62). The impact of maternal near-miss events on poor pregnancy outcomes was worsened when women had delay in seeking care (AOR = 2.9, 95% CI: 1.38–6.20), delay in receiving treatment (AOR = 6.4, 95% CI: 1.50–27.54), mode of transport (AOR = 2.6, 95% CI: 1.21–5.50), and mode of delivery (AOR = 3.5, 95% CI: 1.76–7.04) (Table 3).

## 4. Discussion

The study is aimed at revealing the incidence of adverse perinatal outcomes in women exposed to maternal near-misses. Accordingly, the incidence of adverse perinatal outcomes was found to be 56% in exposed women and 16% in nonexposed women. The exposed group was two times more likely to develop adverse perinatal outcomes than the nonexposed group (AOR = 2.25, 95% CI: 1.10–4.62). This finding is comparable to the previous studies done in Addis Ababa [14], Southeast [17], and Tigray of Ethiopia [20], Rwanda [24], and Nigeria [15]. In the presence of maternal near-miss causes such as hypertension, obstetric hemorrhage, prolonged labour, uterine rupture, anemia, and sepsis, adverse perinatal outcomes are inevitable.

The exposed women who were delayed in seeking obstetric care were nearly three times more likely to have negative pregnancy outcomes than the nonexposed women (AOR = 2.9, 95% CI: 1.38–6.20). Prior studies done in Brazil [21, 25], Nigeria [15], and Ethiopia [26, 27] acknowledged comparable results. Lack of ambulance may explain this finding, less than half 153 (47%) of women utilized an ambulance as the mode of transportation. This may be because obstetric complications, if not diagnosed and treated promptly, can progress to severe forms, which were the cause of adverse perinatal outcomes. Women with maternal near-misses should not be hesitant to seek care as soon as possible. We also suggest that the healthcare providers advise a pregnant woman to seek care as soon as she gets pregnant. Therefore, improving women's timely health-seeking behavior is critical for reducing adverse perinatal outcomes.

The study also found that exposed women who did not travel by ambulance were more than twice as likely as nonexposed women to have adverse perinatal outcomes (AOR =2.6, 95% CI: 1.21–5.50). This finding is supported by studies done in different parts of Ethiopia [6, 7, 28, 29] whereas no similar findings have been found in other countries. Negative perinatal outcomes worsen when labouring women do not arrive at the hospital on time due to traffic congestion. The exposed women traveling by ambulance, on the other hand, may have a better chance of receiving lifesaving treatment on the way to the hospital. Hence, we suggest that the concerned stakeholders increase the number of ambulances available and or/prioritize labouring women before complications arise.

In this study, exposed women who were treated after half an hour (faced delay three) in the hospital were more than six times more likely to have adverse perinatal outcomes than nonexposed women (AOR = 6.40, 95% CI: 1.5–27.54). This finding is similar to studies done in Malaysia [30], Brazil [25], India [31], Mozambique [32], and Ethiopia [33] in that adverse perinatal outcomes were highly linked to third delay. This could be explained by the dearth of qualified and skilled personnel, inadequate staff, limited supply of medicine and equipment, poor facility situations, and poor attitudes and care on the part of healthcare workers which might be all possible explanations for the third delay, and interested parties working on the maternal and neonatal health should prioritize overcoming these impediments. We strongly suggest that the third delay calls on the moral perceptions and ethical responses of healthcare providers to optimize the care provided to women, based on this finding.

The exposed women who had spontaneous vaginal delivery were nearly four times more likely to have adverse perinatal outcomes than the nonexposed women (AOR = 3.5, 95% CI: 1.76–7.04). This finding is comparable to the study done by Brazil [34] and Ethiopia [11] but contrary to studies done by other parts of Ethiopia [35], Brazil [36], and Nigeria [15] reporting that women who delivered by elective caesarean section and forceps developed both neonatal and maternal near-misses. Exposed women who gave birth via SVD were more likely to have adverse perinatal outcomes. This was exacerbated by inadequate monitoring of women during labour and delivery, due to the misleading phrase “normal labour.” This implies that healthcare providers should strive to reduce negligence caused by misunderstanding of “normal labour.” We suggest that no labour be considered “normal labour” because no one predicts complications that arise during labour and delivery [8].

## 5. Limitations of the Study

For reasons of logistic and achievability issues, the study failed to account for some important perinatal outcomes, such as neonatal intensive care unit admittance and neonatal mortality and other among women exposed to the maternal near-misses. We used the one-minute Apgar score. The study was only conducted in public hospitals without including private hospitals providing obstetric services. Therefore, the result of adverse perinatal outcomes does not constitute the bigger population group in the zone.

## 6. Conclusion

The incidence of adverse perinatal outcomes was higher among women exposed to maternal near-misses than the nonexposed. Women who experienced maternal near-miss complications during pregnancy and delivery had a higher incidence of adverse perinatal outcomes due to delay in seeking care, transport mode used, delay in receiving treatment, and mode of delivery used. Therefore, we recommend that increasing the number of ambulance and/or counseling women to utilize maternity waiting rooms ahead of onset of labour to minimize the delays can improve perinatal outcomes. A qualitative study that includes the perspectives of healthcare providers is also required to deal with the third delay.

## Figures and Tables

**Table 1:** Selected variable distribution among exposed and nonexposed women at Arsi Zone public hospitals in Ethiopia, 2022.

Variables	Categories	Exposed group		Nonexposed group		Chi-square test
		*N*	%	*N*	%	
Place of residence	Rural	39	35	122	57	**0.001**
	Urban	73	65	92	43	
Marital status	Married	110	98	205	96	
	Unmarried	2	2	9	4	0.855
Educational level	Illiterate	11	10	32	15	
	Primary	58	52	59	28	0.049
	Secondary	36	32	84	39	
	Tertiary	7	6	39	18	
Interpregnancy interval	<24 months	58	66	69	49	0.135
	>24 months	30	34	72	51	
Received ANC	Yes	100	89	204	95	
	No	12	11	10	5	**0.039**
Number of ANC contacts received	<4	24	24	122	60	**0.001**
	≥4	76	76	82	40	
Delay in seeking care	Yes	70	63	179	84	**0.001**
	No	42	37	35	16	
Delay in arrival at hospital	>1 hr	66	59	66	31	**0.001**
	<1 hr	46	41	148	69	
Delay in getting treatment	<30 minutes	102	91	206	96	
	>30 minutes	10	9	8	4	**0.001**
Mode of transport	Ambulance	71	63	82	38	
	Others	41	37	132	62	**0.001**
Mode of delivery	SVD	40	38	133	62	
	Others	65	62	81	38	**0.001**
Length of hospital stay in days	<5 days	81	72	206	96	
	>5 days	31	28	8	4	**0.001**

Bold: *p* value < 0.05.

**Table 2:** The magnitude of poor perinatal outcomes among exposed and nonexposed women at Arsi Zone public hospitals in Ethiopia, 2022.

Variables of interest	Women lacking near-miss events (*n* = 214)		Women having near-miss events (*n* = 112)		*p* value	95% CI (OR)
	*N*	%	*N*	%		
Adverse perinatal outcomes	34	16	63	56	**0.001**	**6.81 (4.0–11.5)**
Stillbirth	1	0.5	25	22	**0.001**	**9.5 (6.6–13.2)**
Low birth weight	6	3	15	13	**0.001**	**6.4 (4.0–9.7)**
Birth asphyxia	23	11	10	9	0.605	1.23 (0.6–2.7)
Preterm birth	4	2	13	12	**0.001**	**5.2 (3.1–8.2)**

Bold: *p* value < 0.05.

**Table 3:** Logistic regression analysis model depicting the likelihoods of poor pregnancy outcomes among exposed women at Arsi Zone public hospitals in Ethiopia, 2022.

Variables	Categories	Adverse perinatal outcomes		*p* value
		95% CI (COR)	95% CI (AOR)	
Maternal near-miss	Yes	**6.8 (4.0–11.5)**	**2.25 (1.10–4.62)**	**0.028**
	No	1	1	
Number of ANC contacts made	<4	**3.96 (2.24–7.01)**	1.6 (0.77–3.40)	0.210
	≥4	1	1	
Delay in seeking care	Yes	**4.6 (2.66–7.85)**	**2.9 (1.38–6.20)**	**0.005**
	No	1	1	
Delay in arrival at hospital	<1 hr	1	1	
	>1 hr	**3.32 (2.0-5.43)**	0.9 (0.42–1.84)	0.740
Delay in receiving treatment	<30 minutes	1	1	
	>30 minutes	**6.9 (2.4–20.0)**	**6.4 (1.50–27.54)**	**0.012**
Mode of transport used	Ambulance	1	1	
	Others^∗^	**3.9 (2.4–6.6)**	**2.6 (1.21–5.50)**	**0.015**
Mode of delivery	SVD	**5 (2.9–8.8)**	**3.5 (1.76–7.04)**	**<0.001**
	Others^a^	1	1	
Duration of hospital stay in days	<5 days	1	1	
	>5 days	**5.3 (2.6–10.8)**	0.42 (0.16–1.12)	0.082

^∗^Personal vehicles, public transport, Bajaj, and cart. ^a^Assisted vaginal delivery, caesarean section, and vacuum extraction. Bold: *p* value < 0.05.

## Data Availability

The dataset used in this study will be made available upon reasonable request from the corresponding author.

## Abbreviations

ANC: Antenatal care
AOR: Adjusted odd ratio
CI: Confidence interval
COR: Crude odd ratio
C/S: Caesarean section
MNM: Maternal near-miss
SVD: Spontaneous vaginal delivery
WHO: World Health Organization.
